# Supplementary figures and images for: Exogenous applications of brassinosteroids promote secondary xylem differentiation in Eucalyptus grandis
Source: PeerJ. 2024 Jan 2;12:e16250. doi: 10.7717/peerj.16250 (PMC10768668; doi:10.7717/peerj.16250)

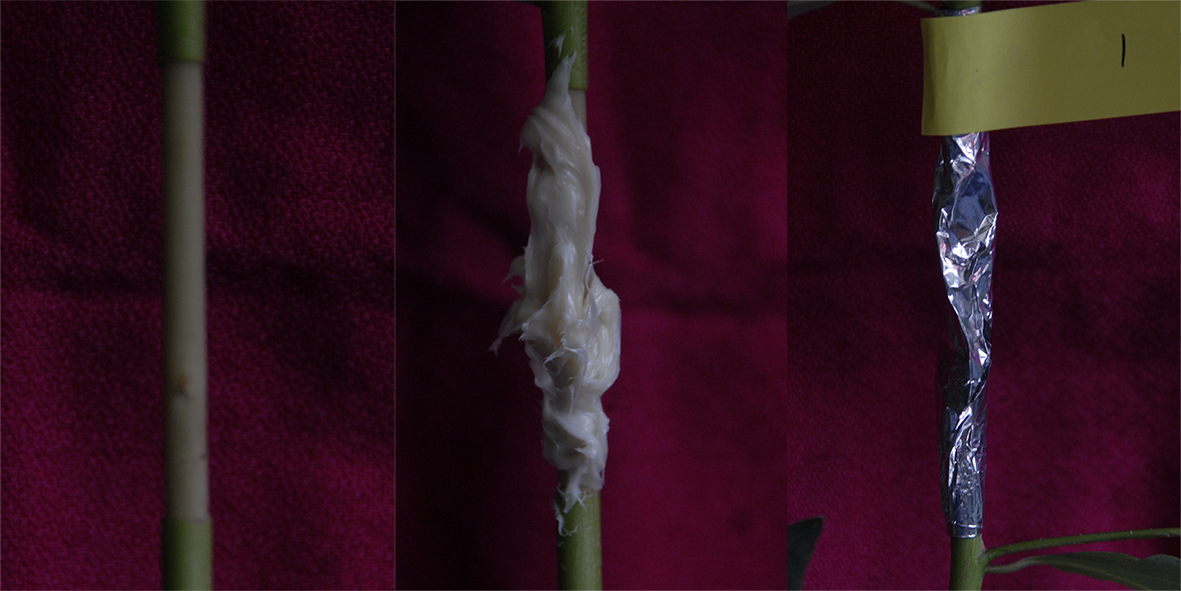

Supplement: Figure S1 [file peerj-12-16250-s001.tif]

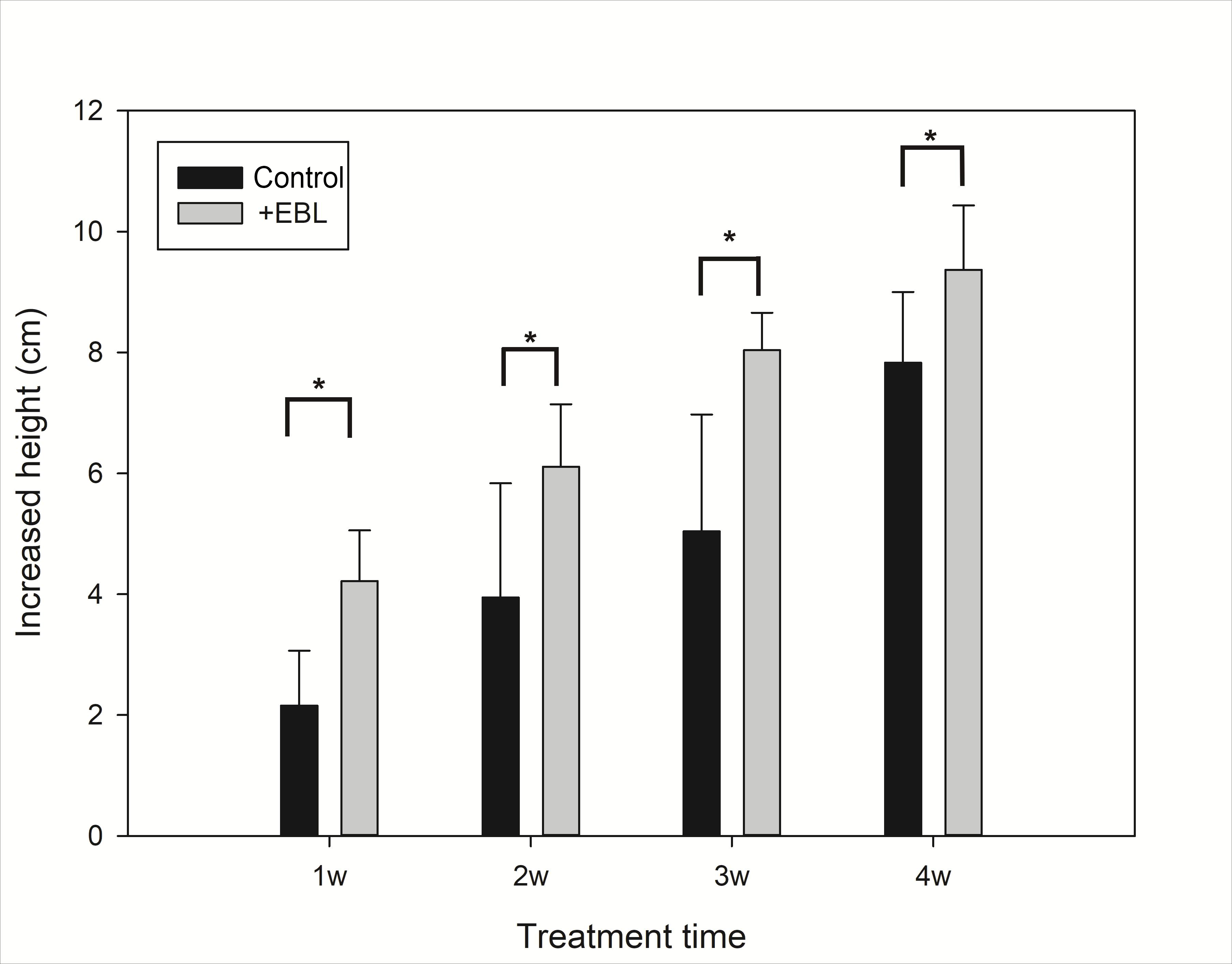

Supplement: Figure S2 [file peerj-12-16250-s002.tif]
